# Prevalence and barriers to early initiation of breastfeeding among urban poor full-time readymade garments working mothers: a mixed-methods study in Bangladesh

**DOI:** 10.1186/s13006-024-00645-w

**Published:** 2024-06-18

**Authors:** Md. Rabiul Islam, Tasnim Tamanna, Nusrat Azrin Mohsin, Arifa Farzana Tanha, Nusrat Hossain Sheba, JMA Hannan

**Affiliations:** 1 Department of Public Health, School of Pharmacy and Public Health, Independent University (IUB), Dhaka, Bangladesh; 2 International Centre for Diarrhoeal Disease Research, Bangladesh (icddr,b), Dhaka, Bangladesh; 3 Department of Public Health, Northern University Bangladesh (NUB), Dhaka, Bangladesh; 4 Department of Pharmacy, School of Pharmacy and Public Health, Independent University (IUB), Dhaka, Bangladesh

**Keywords:** Early initiation of breastfeeding, Readymade garment, Mother, Female worker, Bangladesh

## Abstract

**Background:**

Early initiation of breastfeeding is the initiation of breastfeeding within one hour of birth, which plays a significant role in a born baby’s growth and survival, however its prevalence and predictors among urban full-time readymade garments (RMG) working mothers are not investigated. The purpose of this study is to determine the prevalence and factors affecting early initiation of breastfeeding among urban RMG working mothers.

**Methods:**

A sequential explanatory mixed-methods study was conducted between March 2023 and December 2023 in Dhaka, Bangladesh. A total of 452 full-time female RMG workers were included for the quantitative study. Qualitative study was carried out among 30 full-time female RMG workers, four female physicians who were employed in the RMGs, four RMG factory managers, and four local pediatricians.

**Results:**

The prevalence of early initiation of breastfeeding was 40% among the women. It was significantly associated with various factors, including socio-cultural barriers, the advanced age of the mother (AOR 3.93, 95%CI 1.18, 13.04), lack of education (AOR 6.86, 95%CI 1.11, 42.49), lack of awareness, and cultural practices such as initiating goat milk and honey instead of breast milk. The absence of colostrum feeding (AOR 8.96, 95%CI 4.30, 18.70) and pre-lacteal feeding (AOR 0.06, 95%CI 0.03, 0.11) were significant baby feeding practice-related barriers to early initiation of breastfeeding. Maternal health factors, notably post-delivery sickness, cesarean delivery, and lack of breastmilk production, were revealed as a significant hindrance to the early initiation of breastfeeding explored from qualitative analysis. In addition, RMG factory-related factors that significantly affect early initiation of breastfeeding include a strong focus on production, a busy schedule, and a lack of initiative regarding the early initiation of breastfeeding.

**Conclusions:**

The prevalence of early initiation of breastfeeding among RMG working women is poor. This study emphasizes the need for interventions that address specific challenges of early initiation of breastfeeding faced by working mothers in RMG sectors, including improved lactation education, increased awareness to mitigate cultural barriers, RMG factory-based initiatives to empower female workers early initiation of breastfeeding, and preparing early initiation of breastfeeding -friendly post-cesarean unit at the health care facility.

## Background

Approximately three million infants in the world die every year in their first month of birth, and early initiation of breastfeeding could potentially prevent 22.3% of these deaths [1]. Breast milk is the best formula for the newborn; therefore, to ensure effective breastfeeding, the World Health Organization (WHO) recommends early initiation of breastfeeding within an hour of delivery [2, 3]. It provides necessary nutrients to the newborn in the form of colostrum (first milk), which enhances the immune system, growth factor, and other protective factors [4]. According to a recent systematic review and meta-analysis, the risk of neonatal mortality doubles when breastfeeding is started beyond the first hour of life [5], and the risk of death increases as the initiation of breastfeeding duration increases.

The prevalence of early initiation of breastfeeding is approximately 42% worldwide [1]. While the prevalence is 43% in European nations [6], it varies from 34.7 to 87.2% in African countries [7–12] and 38.7–42% in Asian countries [9, 10, 13, 14]. In low-and middle-income countries, early initiation of breastfeeding practice is minimal [15]. Tarly initiation of breastfeeding rates in several South Asian nations are among the lowest in the world; in Pakistan, India, Bangladesh, and Nepal, they are around 29%, 41%, 47%, and 45%, respectively [16]. Numerous factors have been linked to the early onset of breastfeeding, such as the age, profession, and culture of the mother, as well as prenatal care and lactation, delivery location, delivery method, and number of children [17, 18]. Global data shows that mothers who gave birth in health facilities in Tanzania and Nigeria had 1.5–2 times higher odds of experiencing early initiation of breastfeeding within the first hour of life than those who gave birth at home [8, 12]. The primary factor for the delayed onset of breastfeeding after a cesarean section is the mode of delivery [19]. Numerous studies have indicated that one of the main reasons for inappropriate or delayed initiation of breastfeeding is introducing pre-lacteal food and rejecting colostrum after delivery [20–22]. Prior research indicates a substantial correlation between early initiation of breastfeeding and clinical parameters such as intended pregnancy [23], average birth weight [24], and antenatal care (ANC) [14, 23, 25]. Despite the importance of early initiation of breastfeeding, Bangladesh has consistently had a low incidence of it compared to neighbouring nations [25].

In this era of globalization, women play both productive and reproductive roles, and in Bangladesh, the proportion of female employees has been rising continuously [26]. The International Labor Organization estimates that 18.1 million women were employed in Bangladesh in 2017; of them, rural women made up more of the labor force (37.6%) than urban women (30.8%) [27, 28]. According to reports from 2021, in Bangladesh, 54% of the female workers in the readymade garments (RMG) industry are of childbearing age [29]. However, they have limited opportunities to breastfeed their children due to long work hours, a lack of childcare facilities, inadequate training for lactating women, short breaks, and a lack of private spaces for breastfeeding. Most compliance-based workplaces do not have the necessary facilities for refrigerating pumped breast milk. Although the RMG provides on-site childcare facilities, most do not have a comfortable layout encouraging breastfeeding [30]. As a result, female workers have faced several issues like family crises, a lack of support, disparity, work pressure, stress, and anxiety [31–33]. According to some concurrent studies, only a handful of well-known factories in Bangladesh offer their workers indoor medical services, and a medical staff keeps an eye on and informs pregnant women during the ANC and postnatal care (PNC) periods [33, 34].

Despite several benefits of early initiation of breastfeeding, the women in Bangladesh are not aware of it. Even in RMG factories, where most of the women are urban poor and illiterate, the authority does not take any initiative to increase awareness among their female workers knowledge about the issue. Therefore, the early initiation of breastfeeding proportion is anticipated to be much lower than our national prevalence. To the best of our knowledge, there is no study in Bangladesh and even in the world that determined the causes of the failure of early initiation of breastfeeding among this marginalized group of women. Previously, some studies in some developing countries including Bangladesh found barriers to exclusive breastfeeding, but these studies mainly focused on the clinical features of the mother, omitting their social, occupational and behavioral determinants. Therefore, we assume a research gap in this particular area. This study aims to determine the barriers of the early initiation of breastfeeding among mothers who are working in the RMG sector in Bangladesh. We used a combination of qualitative and qualitative approach to explore the barriers of early initiation of breastfeeding. Through the quantitative study, we have determined the prevalence and barriers of early initiation of breastfeeding. In addition, we have explored in-depth inside of these barriers through the qualitative study. Therefore, this study has comprehensively found the actual scenario of early initiation of breastfeeding and their hindrances among full-time RMG sector working women in Bangladesh.

## Methods

### Study design and population

A sequential explanatory mixed-methods study was conducted among a total of 482 female workers (452 for the quantitative part and 30 for the qualitative part) from four RMG factories in the capital city of Bangladesh. In addition, four female physicians (one from each) working in the factories, four (one from each) RMG factory managers, and four local pediatricians from the adjacent areas were included for the qualitative part of the study to get in-depth insight. The study included adult full-time RMG female workers who had at least one child within one year of age and had worked at least the past two years in the factory. A quantitative method was first used to identify the barriers of early initiation of breastfeeding, and a qualitative approach was used to help extract a better understanding of these barriers. We thus choose a sequential explanatory mixed-methods study [35], in which we applied the quantitative approach initially and the qualitative approach thereafter.

### Integration of quantitative and qualitative approach

We discovered throughout the quantitative survey that several barriers to early initiation of breastfeeding can be further elaborated with the use of a qualitative approach. Then, to explore these barriers further, we developed a qualitative interview protocol and carried out a qualitative study with mothers who worked in the same RMG industries and shared comparable traits. We also considered the views of the relevant stakeholders, namely, female physicians, RMG factory managers, and local pediatricians from the adjacent areas to get in-depth insight. The same data collectors conducted the interviews.

Through a closed-ended data collection questionnaire, we collected quantitative data about the barriers to the early initiation of breastfeeding. With the data, we could not properly clarify our research question. We discovered some variables, most notably the place of birth delivery, colostrum feeding practices, and RMG facility-based barriers, which in-depth interviews (IDI) with the study participants and key informant interviews (KII) with the pertinent stakeholders could further explore.

Therefore, we developed a qualitative interview protocol based on the findings from the initial quantitative phase. This protocol was designed to delve deeper into the barriers to early initiation of breastfeeding by collecting and analyzing qualitative data in the second phase of the study.

We incorporated the results obtained from both the quantitative and qualitative stages while analysing the overall study findings. As indicated, we asked both quantitative and qualitative research questions to better understand the barriers to the early initiation of breastfeeding. In the result and discussion section, we integrated the findings from both study phases to comprehensively address the research questions and establish a stronger and more significant understanding of the research problem.

Initially, we interpreted the results that helped answer the study’s primary quantitative research question: “What are the barriers to early initiation of breastfeeding among RMG female workers?” Then, we discussed the IDI and KII findings, which were aimed at addressing the main research question in the qualitative stage of the study: “How did the selected factors identified in the quantitative phase affect the early initiation of breastfeeding of the RMG female workers?” This process allowed for the findings from the qualitative phase to further clarify and explain the statistical results from the first quantitative phase. Subsequently, we thoroughly discussed the study results by categorizing the findings into the respective quantitative and qualitative research subquestions that pertain to each of the investigated factors influencing the early initiation of breastfeeding. Finally, we enhanced the discussion by referencing relevant literature, encompassing both quantitative and qualitative published studies. Thus, integrating the quantitative and qualitative findings helped in clarifying the findings of the statistical tests, which underscored the purpose of using a sequential explanatory mixed-methods study design to provide more comprehensive insights.

### Sample size calculation and participants selection criteria

#### Selection of factories

We prepared a list of six factories situated in Bangladesh’s capital, Dhaka. Compared to other factories, these factories employed a larger workforce, and a large proportion of the workers were female. In addition, these factories were situated in the heart of the city, which clearly justified the urban poor women included in the title. However, after consulting with the factory managers, two of them did not give us permission due to their busy schedules. We got permission from four factories, and we included them in the study.

#### For quantitative part

The sample size was drawn by using n = z^2^ (pq)/d^2^ formula. Where n is the desired sample size, z is the standard normal deviate (1.96) for a 95% confidence interval, p = estimated proportion (0.66) of outcome variable from the previous study, q = 1-p (0.34), d is the margin of error = 0.05 [24]. After fitting these values in the formula [n = {1.96^2^ × (0.66 × 0.34)}/0.05^2^=0.86/0.0025 = 344.82 ≃ 345}] our calculated sample size was 345. We considered a 10% non-response rate of the participants; therefore, our total sample size turned to 380. The study participants were selected using a multistage sampling method. *Firstly*, we collected a list of a total of 16,000 workers (both genders) from the four factories. Our study was conducted among women only; therefore, a list of 9503 female workers was constructed. *Secondly*, 2412 women who had at least one child were short-listed from these women. *Finally*, the women having a baby under one year of age were considered for the study. Thus, we got a final list of 752 eligible women for our survey. We have included 452 women in this study by using a random sampling technique.

#### For qualitative part

**Fig. 1:**
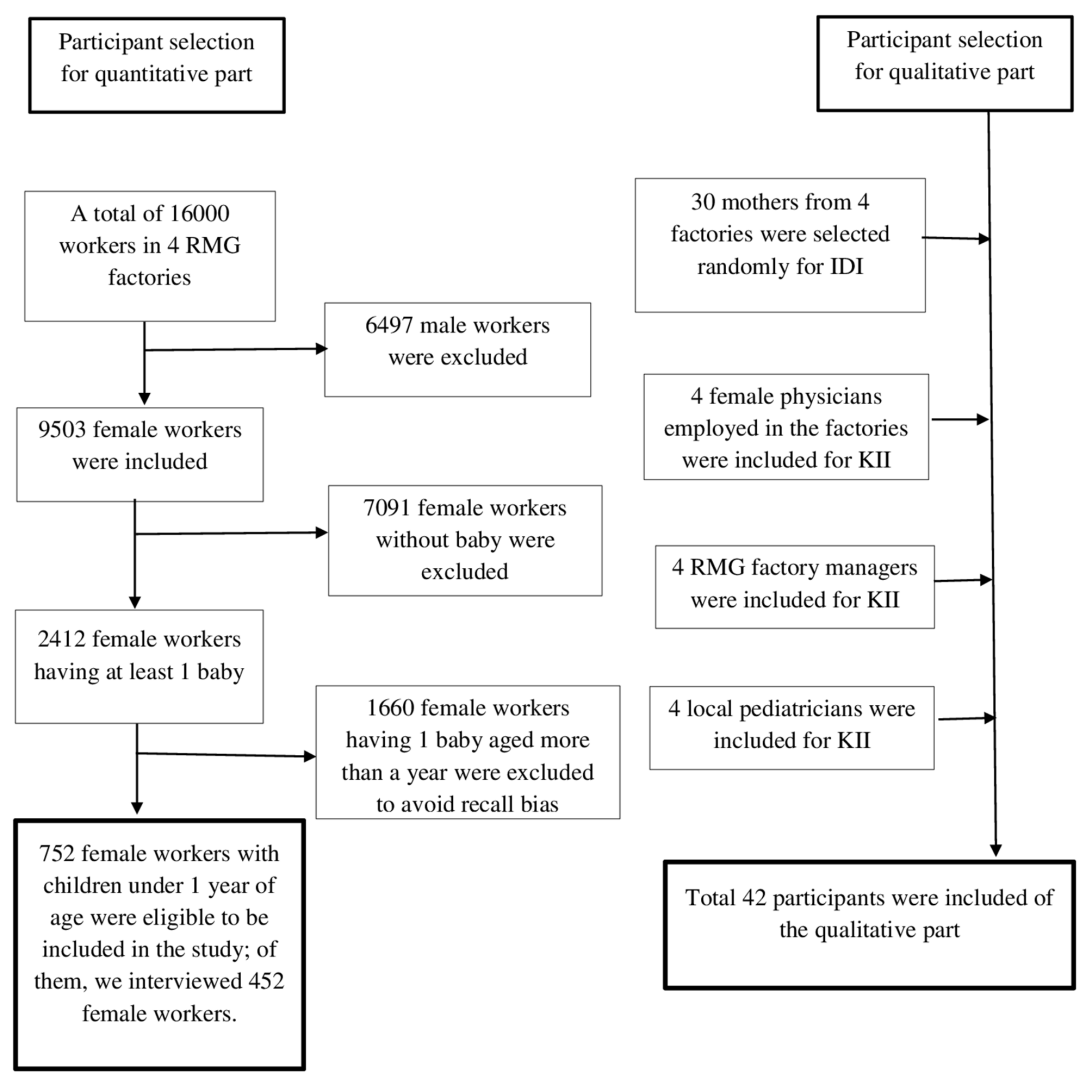
Participant selection for the quantitative and qualitative data collection

A data saturation point was applied to estimate the sample size for IDI and KII of qualitative investigation. When we found the data had been saturated to a point and almost similar responses were coming from all the respondents, we stopped recruiting new respondents. We finally recruited a total of 30 mothers from four factories. All of them were selected randomly. In addition, we randomly included four female physicians (one from each) who were employed in the factory and were the primary contact point for the health problems of the female workers, four RMG factory managers who directly supervised the female workers to explore the barriers to the failure of early initiation of breastfeeding. Most of the women get their services from local pediatricians, so they know the actual scenario of the issue. Therefore, we also randomly selected four pediatricians from the nearest area to explore the barriers to obtain complete insight (Fig. 1).

### Outcome variable

The outcome variable of the study was early initiation of breastfeeding, which was categorized as “≤1 h after birth” or “>1 h after birth”. According to the WHO, when a child is breastfed within one hour of birth, it’s called early initiation; if not done, then it is called late initiation or delayed initiation [36].

### Explanatory variables

Based on the literature review from previous studies [24, 37–39], Age of the mother (18-25yrs, 26-30yrs, 31-45yrs), religion (Muslim, Hindu), Maternal education (Illiterate, Primary, Secondary or Above), maternal body mass index (Normal, Thin, Overweight or obese), monthly family income in thousand Bangladeshi Taka (10,000–20,000, 21,000–30,000, 30,000 or above), head of the family (Male, Female), ANC visit (No ANC, 1–4 ANC, 4 visits or more), breast feeding counseling during ANC (Yes, No), Age of the infant in month (M) (0 to 2 M, 3 to 4 M, 5 to 6 M), infant’s gender (Male, Female), number of children (1st child, 2nd child, 3rd or over), place of delivery (Home delivery, Facility delivery), mode of delivery (Normal, Cesarean section), pre-lacteal feeding (Yes, No), and colostrum feeding (No, Yes), were considered as the explanatory variables for the study.

### Data collection method

#### Quantitative data collection

Data were collected using a semi-structured interviewer-administered questionnaire through a face-to-face interview method. A group of trained data collectors, comprised of three qualified public health specialists and two trained physicians, performed the interviews. There was only one male data collector, and the rest of the data collectors were female because every participant was a woman. The data collectors had three days of training before beginning data collection. Each aspect of potential bias in data collection has been addressed. The same interviewer pre-tested the checklist and data-collection questions to ensure greater understanding. For ease of understanding, the questionnaire was prepared in English and then translated into the local language (Bengali). The interviewers were also trained to avoid asking leading questions to reduce bias. Before the data collection, each participant received information about their autonomy and the objectives of the study. We also trained data collectors on how to build rapport with the RMG women in a friendly manner. Besides we also reassured them that their information would be kept private and anonymous, which encouraged them to be open and truthful. Finally, we trained our data collectors about the cultural sensitivity and privacy of the women. The interview was conducted in Bangla and it was recorded with the permission of the respondents. The data collection tools were validated by both the existing literature and relevant quantitative and qualitative research experts. Before constructing the questionnaire, we did a thorough literature review, from where we set a number of variables to be included in the study. Prior to data collection, we conducted pretesting among 50 RMG mothers with similar characteristics (different from the main study participants). From the pretesting, we edited and corrected the questions and their sequences. Then, we again referred back to test the understandability of the data collection tools and confirmed their reliability.

#### Qualitative data collection

IDI of 30 RMG workers, KII of four female RMG physicians, four RMG factory managers, and four local pediatricians were conducted in the study. All the IDIs and KIIs data were collected by qualitative interview protocol, and they were recorded using a digital recorder during data collection upon their permission. Written informed consent was collected by the interviewers after describing the objectives and purpose of the study.

### Data analysis

We used the statistical software Stata 14 for quantitative data analysis and Microsoft Excel for the qualitative data. Univariate analysis of the variables, including mean, frequency, and percentage, was used as a descriptive statistic. For bivariate analysis, we used the Pearson chi-square test, and the association between the outcome variable and explanatory variables was determined using a binary logistics regression model. For all statistical analyses, a 95% confidence interval (*p* < 0.05) was considered significant. The recorded qualitative interviews were transcribed in English. Each applicable text unit underwent initial open coding using theme analysis methods by two qualitative researchers who separately generated an Excel spreadsheet containing all of the comments provided by the study participants. The final axial coding scheme, commonly acknowledged as an adequate technique for breaking down core themes, was developed by expanding each theme further in light of the themes that emerged from the comments provided by the participants. By manually classifying the text units into themes and sub-themes, two researchers independently applied the axial codes methodically to the data. A comment was marked as “unclear” if its meaning was not clear to both investigators. Any differences of opinion about the theme analysis were resolved by a discussion between the two researchers and a discussion with two senior researchers. The codes and themes were confirmed and discussed at multiple stages of this procedure, and consensus was reached among the members of the research team. Subsequently, it helped us develop an internally validated codebook, which was implemented to encode the data. Finally, the findings are presented in the text.

## Results

### Background characteristics of the respondents

Table 1 illustrates the background characteristics of the mothers who participated in the survey. A total of 452 adult full-time RMG working mothers having at least one child within one year were included in the study. The mean age of the mothers was 25.91 years, and most of them (47.3%) were between 18 and 25 years old. The majority of the study mothers were Muslim (86.1%), primarily educated (60%), had normal BMI (71.2%) and lived in a nuclear family (53.3%). Approximately two-thirds (72%) of the mothers attended the 1 to 4 ANC visits during their pregnancy, whereas 9.0% had no history of ANC Visits, and more than half (55.3%) of the mothers got counseling about exclusive breastfeeding by a healthcare provider during ANC visits.

**Table 1:** Distribution of the characteristics of the study participants (*n* = 452)

Variables	Frequency (*n*)	Percentage (%)
**Age of the mother**		
(Mean ± SD)	25.91(± 3.68)	
18–25	214	47.3
26–30	203	44.9
31–45	35	7.7
**Religion**		
Muslim	389	86.1
Hindu	63	13.9
**Maternal education**		
Illiterate	19	4.2
Primary	271	60.0
Secondary or above	162	35.8
**The history of ANC visit during pregnancy**		
No ANC	42	9.3
1–4 ANC	324	71.7
More than 4 ANC	86	19.0
**Counsel on EBF during ANC visits**		
No	202	44.7
Yes	250	55.3
**Maternal BMI**		
Normal	322	71.2
Thin	87	19.2
Overweight or obese	43	9.5
**Type of family**		
Joint	211	46.7
Nuclear	241	53.3
**Monthly household income (In Thousand Bangladeshi Taka)**		
(Mean ± SD)	24763.27(± 5039.02)	
10,000–20,000	109	24.1
21,000–30,000	278	61.5
30,000 or above	65	14.4
**Head of the family**		
Male	354	78.3
Female	98	21.7
**Age of the baby (in months)**		
(Mean ± SD)	3.49(± 1.06)	
0 to 2 Months	75	16.6
3 to 4 Months	297	65.7
5 to 6 Months	80	17.7
**Gender of the baby**		
Male	240	53.1
Female	212	46.9
**Birth order of the baby**		
1st child	233	51.5
2nd child	166	36.7
3rd or over	53	11.7
**Place of delivery**		
Home Delivery	169	37.4
Facility Delivery	283	62.6
**Mode of Delivery**		
Normal Vaginal Delivery	265	59
Cesarean Section	187	41
**Pre-lacteal feed status**		
No	188	41.6
Yes	264	58.4
**Colostrum feeding status**		
No	171	37.8
Yes	281	62.2
**Early initiation of breast-feeding status**		
≤ 1 h after birth	181	40
> 1 h after birth	271	60

Around 80% of mothers were from patriarchal families whose average monthly family income was 24763.27 thousand Bangladeshi Taka only; the mean age of the infant was 3.49 months, and most of the babies were from the age group 3–4 months (65.7%). According to the birth order, more than half (53.1%) of the babies were female, and 51.5% were the first child. It was also evident that 62.6% of pregnant mothers had received hospital facilities during their delivery, and 41.4% got cesarean section, meaning that 58.6% of pregnant mothers had given birth through expected normal vaginal delivery. More than half (58.4%) of the babies had a history of pre-lacteal feeding (such as honey, formula milk, cow milk, and dates).

Only 40% of the babies got breastfed within one hour after birth, whereas the majority (60%) were delayed in early initiation of breastfeeding. Our qualitative findings from the IDI of the mothers revealed that 16 out of 30 mothers (corresponds to 37%) did not initiate breastfeeding within one hour of their delivery.

### Distribution of early initiation of breastfeeding by the explanatory variables

Table 2 shows the association between mother characteristics and the early initiation of breastfeeding using Chi-square analyses. The results revealed that the failure of early initiation of breastfeeding was significantly higher among illiterate women (*p* = 0.003) and middle socio-economic class women (*p* = 0.014). Besides, the women who delivered their babies in the health care facility are more (65.4%) susceptible to the failure to breastfeed their babies within one hour of delivery (*p* = 0.002).

**Table 2:** Distribution of early initiation of breastfeeding according to the characteristics of the mother

Variables	Early initiation of breastfeeding		*P* value*
	≤ 1 h after birth (%)	> 1 h after birth (%)	
**Age of the mother**			0.801
18–25 years	41.6	58.4	
26–30 years	38.9	61.1	
31–45 years	37.1	62.9	
**Religion**			
Muslim	40.9	59.1	0.371
Hindu	34.9	65.1	
**Maternal education**			0.003
Illiterate	10.5	89.5	
Primary	37.6	62.4	
Secondary or above	47.5	52.5	
**ANC visit during pregnancy**			0.960
No ANC	38.1	61.9	
1–4 ANC	40.1	59.9	
More than 4 ANC	40.7	59.3	
**Counsel on EBF during ANC visit**			0.864
No	39.6	60.4	
Yes	40.4	59.6	
**Maternal BMI**			
Normal	39.8	60.2	0.838
Thin	39.1	60.9	
Overweight or obese	44.2	55.8	
**Type of family**			0.386
Joint	42.2	57.8	
Nuclear	38.2	61.8	
**Monthly household income (In thousand Bangladeshi Taka)**			0.014
10,000–20,000	51.4	48.6	
21,000–30,000	35.3	64.7	
30,000 or above	41.5	58.5	
**Head of the family**			0.772
Female	38.8	61.2	
Male	40.4	59.6	
**Gender of baby**			0.326
Male	37.9	62.1	
Female	42.5	57.5	
**Birth order of the baby**			0.946
1st child	40.3	59.7	
2nd child	39.2	60.8	
3rd or over	41.5	58.5	
**Place of delivery**			0.002
Home delivery	40.1	50.9	
Facility delivery	34.6	65.4	
**Mode of delivery**			< 0.001
Normal vaginal delivery	50.2	49.8	
Cesarean section	25.7	74.3	
**Received postnatal care service**			0.013
No	35.2	64.8	
Yes	46.8	53.2	
**Pre-lacteal feed**			< 0.001
No	77.1	22.9	
Yes	13.6	86.4	
**Colostrum feeding**			< 0.001
No	8.2	91.8	
Yes	59.4	40.6	

*The Pearson chi-square test was performed for the categorical variables

Statistically significant at *p* < 0.05

Similarly, failure of early initiation of breastfeeding practice was found to be considerably higher among the mothers who experienced cesarean section delivery (74.3%) (*p* < 0.001) and who provided any pre-lacteal foods (86.4%) (*p* < 0.001) to their newborns other than breast milk.

Findings from IDI explore that one of the main reasons for not initiating breastfeeding within one hour was the sickness of the mother from post-surgical complications. A RMG working mother (RMG-WM) reported that:

*I had to undergo a cesarean delivery in the hospital. After delivery, I was sick and had to stay in the post-operative observation room. By the time they handed me the baby, four hours already passed; therefore, I could not start early initiation of breastfeeding.* RMG-WM1.

Furthermore, a few of them gave the child formula milk from the very first day after delivery because of their sickness and lack of breast milk production.

*Due to having complications after cesarean section, as I could not put the baby to suck, my mother-in-law gave sugar water to the baby. Even after three days, I had to continue it along with breast milk because of the lack of my breast milk production*. RMG-WM2.

In addition, the decreasing consumption of colostrum feeding showed inversely proportionate to the increasing delayed initiation of breastfeeding (91.8%) (*p* < 0.001) of the study participants.

### Determinants of the failure of early initiation of breastfeeding of the RMG mothers

After adjusting all the co-variates in the logistic regression model, the study determined that the chance of failure of early initiation of breastfeeding was higher among older mothers aged 31–45 years (AOR: 3.93, 95%CI 1.18, 13.04) and illiterate mothers (AOR: 6.86, 95%CI 1.11, 42.49) (Table 3). The study revealed about nine times (AOR 8.96, 95%CI 4.30, 18.70) higher likelihood of not practicing early initiation of breastfeeding for the mothers who did not start colostrum feeding to their baby after their delivery.

**Table 3:** Determinants of the failure of early initiation of breastfeeding

Variables	COR*	95% CI		*P* value	AOR*	95% CI		*P* value
		Lower limit	Upper limit			Lower limit	Upper limit	
**Age of the mother**								
18–25 years	Reference				Reference			
26–30 years	1.110	0.76	1.65	0.578	2.440	0.65	9.19	0.185
31–45 years	1.200	0.58	2.52	0.620	3.930	1.18	13.04	0.025
**Maternal education**								
Secondary or above	Reference				Reference			
Primary	1.500	1.01	2.22	0.044	1.291	0.70	2.40	0.419
Illiterate	7.700	1.72	34.41	0.008	6.860	1.11	42.49	0.038
**ANC visit during pregnancy**								
More than 4 ANC	Reference				Reference			
1–4 ANC	1.020	0.63	1.66	0.923	1.393	0.65	2.98	0.392
No ANC	1.110	0.52	2.38	0.778	0.964	0.25	3.67	0.957
**Counsel on EBF during ANC visit**								
Yes	Reference				Reference			
No	1.030	0.71	1.51	0.864	1.196	0.64	2.24	0.575
**Maternal BMI**								
Overweight or obese	Reference				Reference			
Normal	1.230	0.64	2.36	0.531	1.371	0.56	3.35	0.487
Thin	1.240	0.59	2.60	0.568	1.584	0.53	4.71	0.408
**Type of family**								
Nuclear	Reference				Reference			
Joint	0.840	0.58	1.23	0.386	1.004	0.57	1.78	0.989
**Monthly household income (In Thousand Bangladeshi Taka)**								
30,000 or above	Reference				Reference			
21,000–30,000	1.300	0.75	2.27	0.344	1.408	0.57	3.49	0.460
10,000–20,000	0.670	0.36	1.25	0.210	0.523	0.18	1.49	0.224
**Head of the family**								
Female	Reference				Reference			
Male	1.070	0.68	1.69	0.772	0.857	0.42	1.73	0.666
**Gender of baby**								
Male	Reference				Reference			
Female	0.820	0.57	1.21	0.326	1.234	0.69	2.20	0.476
**Birth order**								
3rd or over	Reference				Reference			
2nd baby	1.100	0.59	2.07	0.761	0.631	0.21	1.86	0.403
1st baby	1.040	0.57	1.92	0.876	0.893	0.28	2.90	0.850
**Place of delivery**								
Home delivery	Reference				Reference			
Facility delivery	1.820	1.24	2.69	0.002	0.784	0.39	1.59	0.499
**Mode of delivery**								
Normal vaginal delivery	Reference				Reference			
Cesarean section	2.910	1.94	4.38	< 0.001	0.708	0.33	1.53	0.380
**Received postnatal care service**								
Yes	Reference				Reference			
No	0.618	0.42	0.91	0.014	1.280	0.66	2.51	0.459
**Pre-lacteal feeding**								
Yes	Reference				Reference			
No	0.040	0.03	0.08	< 0.001	0.060	0.03	0.11	< 0.001
**Colostrum feeding**								
Yes	Reference				Reference			
No	16.420	9.05	29.82	< 0.001	8.960	4.30	18.70	< 0.001

A Binary logistic regression test was performed

*COR: Crude odds ratio

**AOR: Adjusted odds ratio (Adjusted for all the variables presented in the table)

Statistically significant at p ≤ 0.05

The qualitative analysis showed that 7 of 11 mothers who did not start early initiation of breastfeeding failed to start colostrum feeding their baby. A mother was found during IDI whose family insisted on giving Goat milk instead of breast milk to make the baby stronger even though the mother had enough production of breast milk at that time. She uttered that:

*My Father-in-law believed that goat milk would make my baby much stronger; that’s why he insisted on it*. RMG-WM3.

Another mother stated that:

*My mother suggested I give honey instead of breast milk to my baby just after delivery. She heard from neighbors that if a baby is given something sweet after delivery, he/she will learn to talk with a lovely voice.* RMG-WM4.

On the other hand, the mother who did not give any pre-lacteal feeding (AOR: 0.06, 95%CI 0.03, 0.11) found less chance of the failure of early initiation of breastfeeding than the mother who had a history of pre-lacteal feeding. To be more specific, if a mother gave pre-lacteal foods (other than breast milk) to their baby, they significantly failed the early initiation of breastfeeding to their baby. KII of the local pediatricians, RMG residential doctors, and RMG factory managers explored superstition, lack of education, lack of awareness, inadequate knowledge, sickness after delivery, insufficient production of breast milk, lack of ANC visits, cultural factors, highly production-oriented busy schedule, are the key barriers of early initiation of breastfeeding among this lower socio-economic group working mothers.

A mother reported that:

*I have visited the doctor four times during my pregnancy, and I delivered my baby to a hospital. The midwives and doctors suggested I give the first milk to my child just after birth, and I did so.* RMG-WM5.

Another mother said that:

*I have been working in the RMG for the last 13 years. There is a medical center at the factory. They provide some ANC but not up to the mark for us. I could go to the doctor during my pregnancy since I do not have enough money to pay. In addition, I must maintain a very tight schedule in my job. Even we got only two months of maternity leave, which is not enough.* RMG-WM6.

### Findings from the KII of the local pediatrician (LP)

A local pediatrician stated that:

*I have been practicing for the last 27 years. I always suggested mothers to initiate breast milk within one hour after their delivery. However, mothers are more interested in listening to their relatives. They even give some other foods like water, honey, goat milk, cow milk, and dates to their newborn even though they have enough breast milk. I think they do not know the importance of giving breast milk within one hour after delivery. These problems are more common among the illiterate and poor women.* LP1.

Another KII of a pediatrician depicted that:

*I can tell you the scenario of the mothers who give birth in the hospitals only. In my experience, sometimes the cesarean mother becomes too sick to give their colostrum to their baby. In addition, a shortage of breast milk production is another reason for not initiating breast milk within one hour. However, the actual scenario of the mother who gave birth at home is unknown to me.* LP2.

### Findings from the KII of the female physicians (FP) employed in the factories

A female doctor working in the RMG factory pointed out that:

*Majority of the workers are poor and uneducated. Generally, they are migrants from different parts of the country and live in the nearby slums. Therefore, they have cultural diversity. Truly speaking, in RMG, we provide treatments for minor ailments like diarrhea, headache, gynecological problems, and common cold. We have some facilities for baby care, and ANC but we have no facility for the women to ensure their early initiation of breastfeeding. We often found babies with common colds or diarrhea here, and after taking history, maximum times it shows that they were not breastfed. So, it’s a burning issue for the child health of the RMG mother.* FP1.

Similarly, another physician of an RMG factory illustrated that:

*Women come to us during their pregnancy. Sometimes, we suggest they initiate breastfeeding early. But they generally do not pay heed to our advice due to rush hour. We have around 2500 workers, but I am the only doctor. I remain too busy, so I have limited time to give such health education to all of the the mothers in my factory. Another problem is that not all mothers come to us during pregnancy. At the time of delivery, they stay with their family members, and they are more interested in listening to them. They are culturally bound. To me, it is important that a mother must know the benefits of breastfeeding. For RMG sectors mothers, some orientations or training must be arranged.* FP2.

### Findings from the KII of the RMG factory managers (FM)

A manager of a factory said that-.

*We mainly focus on the production. We do not have any capacity or system to educate our female workers about the importance of the early initiation of breastfeeding. We do not even get such initiative from any other health care providers. We allow them maternity leave and we have a lack of control over their early initiation of breastfeeding issue.* FM1.

Another RMG manager uttered that-.

*We have breastfeeding corner and daycare centre, but due to working hours and workload, it is sometimes challenging to maintain its quality. On the other hand, we do not have any concerns about their early initiation of breastfeeding. I do not know about any strategy of the RMG factory to educate these mothers about it. We do have doctors who serve the women and their babies.* FM2.

## Discussion

The early initiation of breastfeeding is the newborn’s first and most crucial immunization for their survival. To the best of our knowledge, this is the first study to investigate the prevalence and barriers of early initiation of breastfeeding among full-time female RMG employees in Bangladesh and the world. Using a sequential explanatory mixed-methods study, we discovered that only 40% of women started breast-feeding within one hour after giving birth. The findings of this study shed light on the challenging nature of working women’s failure to engage in early initiation of breastfeeding. Advanced age of the mother, lack of education, lack of awareness, colostrum feeding practice, pre-lacteal feeding practice, cultural factor, sickness after delivery, insufficient production of breast milk, lack of ANC visits, highly production-oriented busy schedule, and a limited RMG factory initiative were all significant predictors of the failure of early initiation of breastfeeding.

### Prevalence of early initiation of breastfeeding

The present study found that 40% of mothers started nursing within the first hour of birth, lower than the 60.8% reported in the latest Bangladesh Demographic and Health Survey 2018 [38]. The study’s findings are comparable to Nepal (47.3%) [40], higher than India (32%) and Nigeria (34.7%) but lower than Sri Lanka (83.3%) [8, 41, 42]. This suggests that the early initiation of breastfeeding failure prevalence was similar to Nepal, lower than reported in India and Nigeria but higher than in Sri Lanka. A similar prevalence of early initiation of breastfeeding (43.6%) was also found in a study conducted in Saudi Arabia [43]. Such disparity in rates of early breastfeeding within and outside of Bangladesh is most likely related to changes in the mother’s geography, ethnicity, culture, and level of socioeconomic status.

The rate of early initiation of breastfeeding failure among Bangladeshi women is 60%, as reported by the current study. The results of the study showed that childbearing women in Bangladesh are not complying with the breastfeeding recommendations. According to results from a previous study, the prevalence of early initiation of breastfeeding in Bangladesh is 61.19%, while the prevalence of failure in early initiation of breastfeeding was 38.81%, substantially lower than our study prevalence in the case of failing in early initiation of breastfeeding [37]. According to one Nepalese study, 33.6% of mothers failed to initiate breastfeeding within one hour of birth [24], which is lower than the findings of our study (60%). On the contrary, the prevalence rate is lower when compared with previous studies from India (63.6%) [44], and Pakistan (91.5%) [45].

### Factors influencing early initiation of breastfeeding and comparison with previous studies

Comparatively, a lower prevalence of early initiation of breastfeeding was observed among older mothers aged between 31 and 45 years (AOR: 3.93, 95%CI 1.18, 13.04). An earlier study in Bangladesh demonstrates a strong negative correlation between early initiation of breastfeeding and mothers older than 30 years of age (AOR = 0.87, CI = 0.76, 0.99) [23]. In this study, younger women were found to start breastfeeding earlier than older women, which is comparable to a study done in Uganda where a comprehensive analysis revealed that being a young mother (under 34 years old) was strongly associated with initiating breastfeeding in the first hour after birth (OR: 2.00, *p* = 0.001) [46]. This may be due to young people’s higher exposure to schooling than older women [46]. Since educated women tend to be more aware than uneducated women. Likewise, a Namibian study revealed that adolescent girls were more likely to initiate breastfeeding within the first hour after giving birth [47]. However, according to research conducted in Nepal [48] and in Tanzania [49], the risk of delayed breastfeeding was higher among young women. Inexperience and a sense of insecurity among young mothers could be attributed to delayed breastfeeding among young women [49].

Numerous prior studies have shown that maternal education significantly affects early initiation of breastfeeding. Similar to the findings of this study, which showed that illiterate mothers failed to initiate breastfeeding within an hour of delivery (AOR: 6.86, 95%CI 1.11, 42.49), mothers with secondary education [23, 25, 50] and who had attended formal education [17, 44, 51, 52] were more likely to breastfeed their infants earlier than mother with no formal education. Furthermore, our study revealed a positive correlation between mother education and early breastfeeding initiation, similar to earlier findings from India [44] and England [53]. This might be the case as educated women are more conscious of their children’s health since they have better health knowledge [23]. Moreover, educated mothers are better equipped to take in and understand information about health promotion, manage skilled or professional birth aids, and select whether to deliver in a medical facility [54]. In contrast to our findings, a study reveals that lower educated groups generally had a higher rate of early commencement of breastfeeding [38].

This current study reveals that initiation of pre-lacteal feeding leads to failure of early initiation of breastfeeding (AOR: 0.06, 95%CI 0.03, 0.11), which is consistent with findings in a previous study [55]. Pre-lacteal feeding has been identified by 10.3% of mothers as a cause for postponing the initial phase of breastfeeding [43]. In addition, some prior studies reported that pre-lacteal feeding may lead to lactation failure and reduce the newborn suckling response [56–58].

The study revealed that there is about nine time’s higher likelihood of not practicing early initiation of breastfeeding for the mothers who did not start colostrum feeding to their baby after their delivery. The fact that mothers give other pre-lacteal foods (like honey, goat milk, dates) instead of breastmilk. Therefore, the early initiation of breastfeeding failure rate is higher among them.

In order to accomplish the Sustainable Development Goals on lowering mother and child mortality by 2030, institutional delivery promotion is a priority intervention [59]. As a result, the decreasing coverage of early breastfeeding initiation in healthcare facilities is cause for concern. Furthermore, no nation has a breastfeeding rate of more than 80% within an hour of delivery. According to WHO (2015), 42% of babies worldwide receive breast milk within the first hour of life [60]. This consensus states that encouraging and enabling the use of maternal health services must be a top priority in order to advance the cause of early breastfeeding initiation. The findings of this study will assist the government in taking action to promote early commencement of breastfeeding practices in Bangladesh, especially among working women.

### Qualitative insights

The qualitative data analysis depicted the mother’s physical health as a significant barrier to early initiation of breastfeeding. Mothers, especially those who have undergone cesarean section or surgical delivery, are more vulnerable to the failure of early initiation of breastfeeding. Similarly, some previous studies corroborated our findings [61, 62]. They also found that the early initiation of breastfeeding rate is remarkably lower among cesarean mothers. This might be described by the fact that a mother who has undergone cesarean delivery generally keeps away from the baby after delivery, and it is difficult for the mother to recover within one hour after delivery [23]. Cesarean delivery takes place in a hospital or healthcare facility. Therefore, necessary interventions should be taken to prepare the post-caesarean section to facilitate early initiation of breastfeeding.

In addition, there is a lack of skilled birth attendants in developing countries, so it might affect the early initiation of breastfeeding [47]. The study determined a lack of awareness and counselling among RMG workers. Therefore, it is crucial to take a breastfeeding counselling program among the RMG workers. We also found that women are greatly influenced by their family members and society. Hence, community-level breastfeeding awareness is also necessary. The lack of women’s decision-making power is also a significant barrier to early initiation of breastfeeding among mothers. Some prior studies support this finding [39, 63, 64]. Thus, the study urges to improve women’s decision-making capacity. Community-based gender equity programs are needed to educate the people in developing countries.

### Limitations of the study

The study has some limitations. Firstly, it was a cross-sectional study; therefore, we failed to establish a causal association between the barriers of early initiation of breastfeeding and another explanatory variable. Secondly, the possibility of retrospective recall bias of the respondents. It would be preferable to ask mothers about early initiation of breastfeeding immediately after delivery to obtain precise information. But we could not do that. In addition, during the qualitative survey, we focused on the variables included only in the quantitative survey. Therefore, we think that the qualitative data losses its flexibility and depth, which could be a scope of further qualitative study. Finally, the study has been conducted among workers of four factories in central Bangladesh. Therefore, the generalizability of the study findings needs to be used cautiously.

## Conclusions

The study determined increased maternal age, lack of literacy, lack of awareness of feeding colostrum to the baby instead of breastmilk, cultural influence, lack of women’s decision-making power, cesarean delivery, lack of breast milk production, inadequate ANC counseling, heavily production-oriented timetable, limited initiative of the RMG factory are the critical barriers of early initiation of breastfeeding among the lower socio-economic urban RMG working mothers. Some definitive health programs need to be taken to ensure early initiation of breastfeeding among RMG mothers. To start with, an awareness program for the mothers to educate them. They must be informed to avoid conceiving a baby at a later age. In addition, intervention should be taken to prepare the post-cesarean unit as an early initiation of breastfeeding -friendly unit. All the staff can be trained to ensure early initiation of breastfeeding. Furthermore, the RMG factory should take necessary steps to ensure their worker’s baby’s early initiation of breastfeeding, such as employing a dedicated breastfeeding and baby care counselor and reducing working hours for pregnant women to plan for breastfeeding. The governments, public health units, and policymakers can plan some community-based approaches to enhance women’s decision-making and awareness of the community people regarding the importance of early initiation of breastfeeding. Addressing cultural beliefs and misconceptions related to early initiation of breastfeeding is crucial. Mothers should also be made aware that pre-lacteal food needs to be avoided if a mother has enough breast milk for the baby.

## Data Availability

No datasets were generated or analysed during the current study.

## Abbreviations

ANC: Antenatal Care
IDI: In-depth Interview
KII: Key Information Interviews
PNC: Postnatal Care
RMG: Readymade Garments
WHO: World Health Organization
